# Safe model based optimization balancing exploration and reliability for protein sequence design

**DOI:** 10.1038/s41598-025-12568-5

**Published:** 2025-07-29

**Authors:** Shuuki Takizawa, Keita Mori, Naoto Tanishiki, Dai Yoshimura, Atsushi Ohta, Reiji Teramoto

**Affiliations:** Research Division, Chugai Pharmaceutical Co., Ltd, Yokohama, Japan

**Keywords:** Antibody therapy, Machine learning, Protein design

## Abstract

Discovering proteins with desired functionalities using protein engineering is time-consuming. Offline Model-Based Optimization (MBO) accelerates protein sequence design by exploring the vast protein sequence space using a trained proxy model. However, the proxy model often yields excessively good values that are far from the training dataset and causes pathological behavior in the MBO. To address this problem, we propose a mean deviation tree-structured Parzen estimator (MD-TPE) that penalizes unreliable samples located in the out-of-distribution region using the deviation of the predictive distribution of the Gaussian process (GP) model in the objective function to find the solution in the vicinity of the training data, where the proxy model can reliably predict. Upon examining the GFP dataset, compared to TPE, MD-TPE yielded fewer pathological samples. Additionally, it successfully identified mutants with higher binding affinity in the antibody affinity maturation task. Thus, our developed safe optimization approach is useful for protein engineering.

## Introduction

Protein engineering aims to design and discover novel proteins with desirable characteristics. However, this process can be time-consuming and costly because the search space for exploration is very large. Therefore, it is often difficult to use trial and error to determine the best solution. To reduce cost and time, machine learning techniques can be applied using a proxy model trained with the properties of interest and protein sequences^1–3^.

When sufficient samples for training a proxy model are available, a method called offline Model-Based Optimization (MBO) can be used to find a solution that optimizes the objective function^4–6^. In short, MBO is a framework for exploring a vast search space using a proxy model learned from the training data. However, when the input is naively optimized to optimize the proxy function, which is trained on limited data, the proxy model often yields excessively good values for samples that are far from the training dataset. This is because the proxy model, which is usually learned with supervised learning, assumes that test samples are drawn from *i.i.d*, the same distribution as the training data distribution^7–9^. Supervised learning assumes that the training and test samples come from the same distribution. Therefore, it is extremely challenging for standard supervised learning to predict samples shifted from the training data distribution^10,11^. However, in the MBO setting, the proxy model must handle out-of-distribution data that are far from the training data distribution. This causes pathological behavior during the exploration of the search space in MBO^12–19^.

To address this problem, we introduce a novel objective mean deviation (MD) that is optimized to penalize samples in an unreliable region. In our framework, we evaluate the reliability as the uncertainty of the trained Gaussian Process (GP)^20^ model, which is often used as the proxy model in MBO, because the deviation of the GP predictive distribution can capture the uncertainty and quantify the deviation from the training data. MD can explore the search space in the vicinity of the training data, which is a reliable region for the proxy model, by incorporating the deviation and predictive mean of the GP. In this study, we used the tree-structured Parzen estimator (TPE)^21^ to optimize the properties of interest of proteins because TPE can naturally handle categorical variables and protein sequences that consist of amino acids. Subsequently, we proposed TPE with MD as the objective (MD-TPE), which combines TPE and our proposed objective MD, to efficiently explore the protein sequence space while avoiding unreliable regions that are out-of-distribution.

**Fig. 1:**
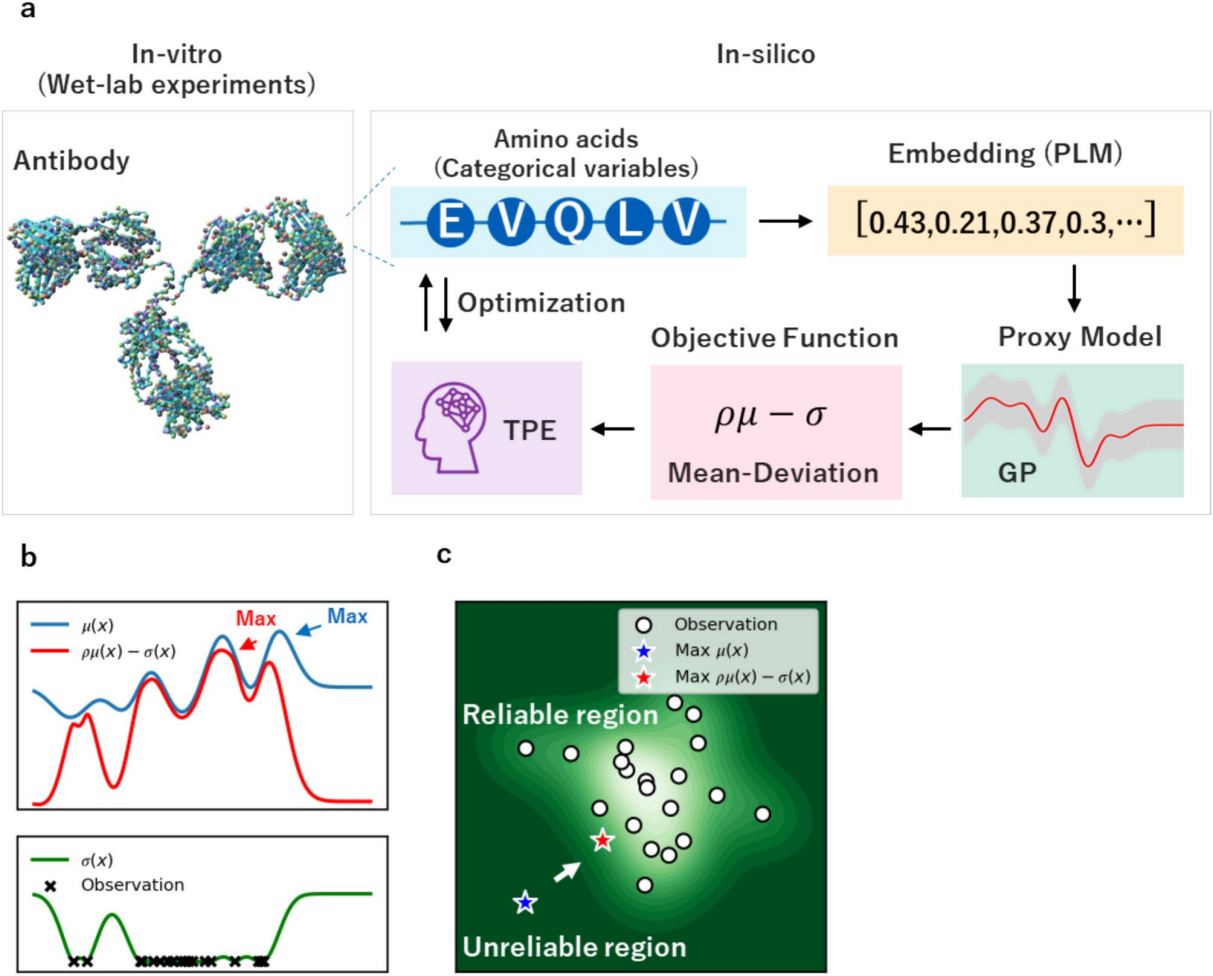
Overview of TPE with mean deviation (MD-TPE). (**a**) Workflow of protein sequence design with TPE with mean deviation (MD-TPE). (**b**) Illustration of the difference of the objective functions between TPE ($\mu \left(x\right)$) and MD-TPE ($\rho \mu \left(x\right)-\sigma \left(x\right)$). (**c**) Illustration of the obtained solutions by TPE and MD-TPE.

Figure 1a illustrates the overall process of the proposed offline MBO framework, MD-TPE. Amino acids were used as categorical variables for the TPE. After embedding the protein sequences into vectors using a protein language model (PLM)^22–24^, we trained the proxy model using GP. Finally, we used MD as the objective function to be optimized. We demonstrated the effectiveness of the GFP dataset^25^ and further validated wet-lab experiments using the antibody affinity maturation setting.

This study focuses on experimentally verifying whether incorporating the predictive uncertainty of Gaussian Process (GP) models as a penalty term in the objective function can mitigate the out-of-distribution (OOD) exploration problem in offline MBO frameworks using Tree-structured Parzen Estimator (TPE). The TPE-based sequence optimization approach is a method that explores sequences incorporating mutations that frequently appear in training data with high value. This differs from de novo generation methods such as CbAS^12^. While some researchers aim to explore “dark” regions of protein space, our emphasis is different—we seek to design reliable protein sequences with high confidence within limited experimental resources, rather than exploratory designs. Gaussian Process models have been widely utilized in protein design within Bayesian optimization frameworks due to their ability to output both predictive values and their associated uncertainties. We propose a method that aims to achieve more reliable sequence design by incorporating this predictive uncertainty as a penalty term in the offline MBO objective function to suppress exploration in OOD regions. We developed Mean Deviation TPE (MD-TPE), which integrates GP predictive uncertainty into a TPE-based offline MBO framework and evaluated the effectiveness of our method through both computational experiments with green fluorescent proteins (GFP) and wet-lab experiments in antibody design.

To summarize, the main contributions of this study are as follows:

1. We demonstrated that MD-TPE safely explored the protein sequence space in the vicinity of the training data.
2. In the GFP brightness task, our method successfully identified brighter mutants than conventional TPE.
3. In the antibody affinity maturation task, we found that our method is indispensable for discovering the expressed proteins from the vast sequence space because antibodies from conventional TPE were not expressed at all.

## Results

### Problem setup and safe exploration for offline model-based optimization

Here, we consider the problem of offline model-based optimization. We encounter oracle f(x), a black box function with discrete inputs $x\in X$, which returns the measured value. In this study, the discrete inputs $x$ correspond to protein sequences, and the discrete space indicates the protein sequence space to be searched. The goal of the standard offline MBO is to find the input x that maximizes the oracle over $X$ as follows:

1 $${x}^{*}:=arg\underset{x\in X}{\text{max}}f\left(x\right)$$

Here, we suppose that the observations of oracle f(x) are given as the accessible static dataset $D=\{\left({x}_{0},{y}_{0}\right),\dots ,\left({x}_{n},{y}_{n}\right)\}$ and additional observations cannot be obtained. The offline MBO algorithm require the proxy function $\widehat{f}(x)$ trained by static dataset D from the oracle f(x). The offline MBO algorithm optimizes proxy function instead of the oracle. When naively solving the above optimization problem, we encounter the problem of overestimating the objective values in the out-of-distribution region because valid inputs $x$ lie on a thin manifold in a high-dimensional space. In general, out-of-distribution proteins lose their function and are not expressed. Therefore, these samples were excluded as much as possible. Consequently, it is desirable to safely search for space in the vicinity of the training dataset, while avoiding out-of-distribution points. To address this problem, taking into account the penalty function $g\left(x\right)$ in the objective function is proposed, as shown below.

2 $${x}^{*}:=arg\underset{x\in X}{\text{max}}\rho \widehat{f}\left(x\right)-g(x)$$

Here, the penalty function $g\left(x\right)$ represents how far the points are from the training data and $\rho$ is the risk tolerance parameter to balance the oracle and the penalty. When $\rho$ > 1, the sampler weights the oracle value more than the penalty function and tends to explore far from the training data. Especially, as $\rho \to \infty$, the problem reduces to the original MBO without the penalty function. In contrast, when $\rho$ < 1, it safely explores in the vicinity of the training data.

As mentioned above, we can avoid the overestimation risk of out-of-distribution by incorporating the penalty function in the objective. However, we usually employ GP ^20,26^ as the proxy model during MBO and can naturally define the deviation of the posterior predictive distribution of the GP model as the penalty function (Supplementary Figure S1). Thus, we propose to use the predictive mean $\mu \left(x\right)$ and the deviation $\sigma \left(x\right)$ of the posterior predictive distribution of GP model as the proxy model $\widehat{f}\left(x\right)$ and the penalty function $g\left(x\right)$ in Eq. (2), respectively. By adopting this approach, the objective in (2) transforms into the mean deviation (MD), which is related to the objective function in portfolio optimization problems^27,28^.

3 $$MD=\rho \mu (x)-\sigma (x)$$

Since $\sigma \left(x\right)$ quantifies uncertainty, MD aims to avoid unreliable out-of-distribution regions during exploring and safely explores the search space in the vicinity of the training data. Figures 1b and 1c illustrate the differences in behavior between the original MBO and MBO with MD. As shown in Fig. 1b, MD finds the optimal solution by incorporating a penalty function. In contrast, the original MBO considers only the proxy model. Figure 1c shows that MBO explores the unreliable region whose uncertainty is large, while MD stays in the reliable region whose uncertainty is small. Regarding the proxy model, alternative models capable of estimating uncertainty, such as the deep ensemble^29^ model and Bayesian neural network^30,31^, can be used.

Several optimization methods such as reinforcement learning and Bayesian optimization (BO) can be used to optimize Eq. (3). Since we aimed to discover desirable protein sequences in this study, we decided to use TPE^21^, a major BO method, as the offline BO method (see details in the Tree-structured Parzen estimator section in Methods). TPE naturally handles categorical variables, such as protein sequences composed of 20 amino acids, and fits our problem setting to optimize their properties. TPE constructs two probability distributions: one from high-performing sequences and another from low-performing sequences. By maximizing the ratio between these distributions, the algorithm preferentially samples amino acid combinations that appear more frequently in successful protein variants. This method effectively captures position-specific amino acid preferences from the training data, guiding the search toward regions with high objective function values of the vast combinatorial sequence space. We refer to the method that uses MD as the objective of TPE MD-TPE.

### MD-TPE showed safe optimization in the GFP brightness task

**Fig. 2:**
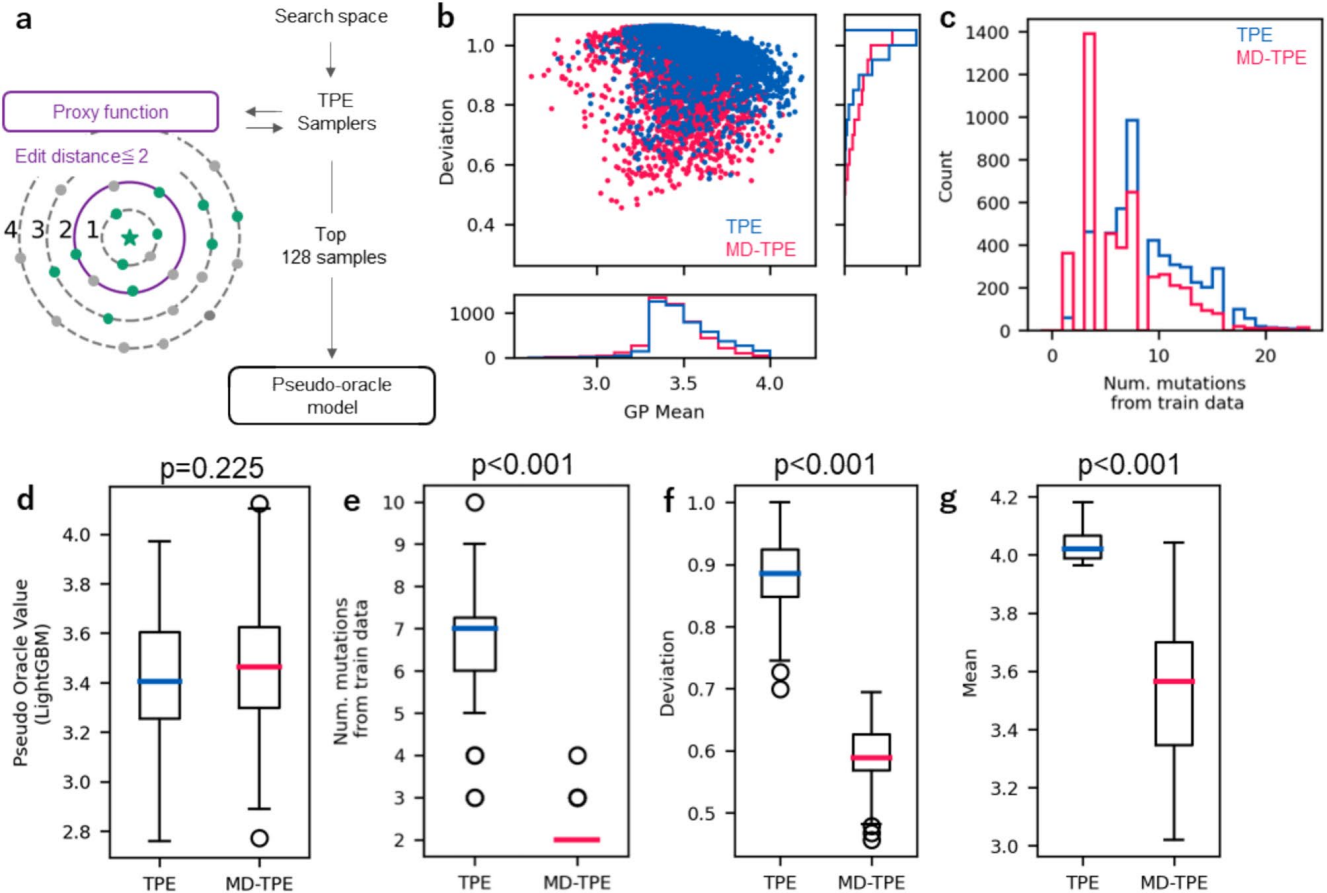
Results of the GFP brightness task. (**a**) Schematic illustration of the workflow for optimization setting and evaluation. (**b**) The relationship between the GP mean and deviation of the sampled value in each run. The use of MD as the objective function yields a low GP deviation (i.e., low uncertainty) through optimization. (**c**) Edit distance from avGFP sequence (i.e., template GFP sequence). (**d-g**) Evaluation for the top 128 proposed sequences from TPE or MD-TPE. (**d**) Distribution of pseudo-oracle values. Mann-Whitney U test did not show significant difference between TPE and MD-TPE groups (p = 0.225) (**e**) Edit distance from template avGFP sequence. Mann-Whitney U test showed significant difference between TPE and MD-TPE groups (p < 0.001) (**f**) GP deviation of proposed sequences. Mann-Whitney U test showed significant difference between TPE and MD-TPE groups (p < 0.001) (**g**) GP mean of proposed sequences. Mann-Whitney U test showed significant difference between TPE and MD-TPE groups (p < 0.001).

First, the safe exploration behavior of MD-TPE was verified using the GFP dataset. To mimic practical molecular optimization process, we limited GFP mutants to those with two or fewer residue substitutions from the parent avGFP sequence as the training dataset for the GP proxy model to investigate the behavior of the samplers (see Fig. 2a and GFP brightness in Methods). Subsequently, GFP brightness was optimized using conventional TPE and MD-TPE. As a result of optimization, MV-TPE successfully explored the sequence space with lower uncertainty (GP deviation) than TPE (Fig. 2b, Supplementary Figure S2), which reflects safe exploration behavior. Moreover, the sequences explored using MD-TPE had fewer mutations from the parent sequence than those explored using TPE (Fig. 2c), indicating that MD-TPE performs safer optimization.

The safe optimization shown by MD-TPE is important because sequences distant from the parent sequence often lose their original characteristics and functions. When the top 128 sequences were evaluated for each condition, MD-TPE identified some sequences with higher pseudo-oracle values. (Fig. 2d), indicating that it identifies promising sequences near the measured sequences through safe optimization. Furthermore, among the top 128 sequences, those sampled by MD-TPE had a maximum of four mutations from the parent sequence (Fig. 2e) and had a smaller GP deviation than that sampled by TPE (Fig. 2f). Moreover, compared to those identified by TPE, MD-TPE identified sequences with a lower GP mean (Fig. 2g). The scatter plots between the GP mean, GP deviation, and TD and pseudo-oracle values are shown in Supplementary Figure S3. These results demonstrate that MD-TPE prevents the selection of overly adventurous sequences that are far from the measured sequences, enabling safe optimization.

### Safe exploration explores reliable region in the anti-MarvelD3 antibody affinity maturation task

**Fig. 3:**
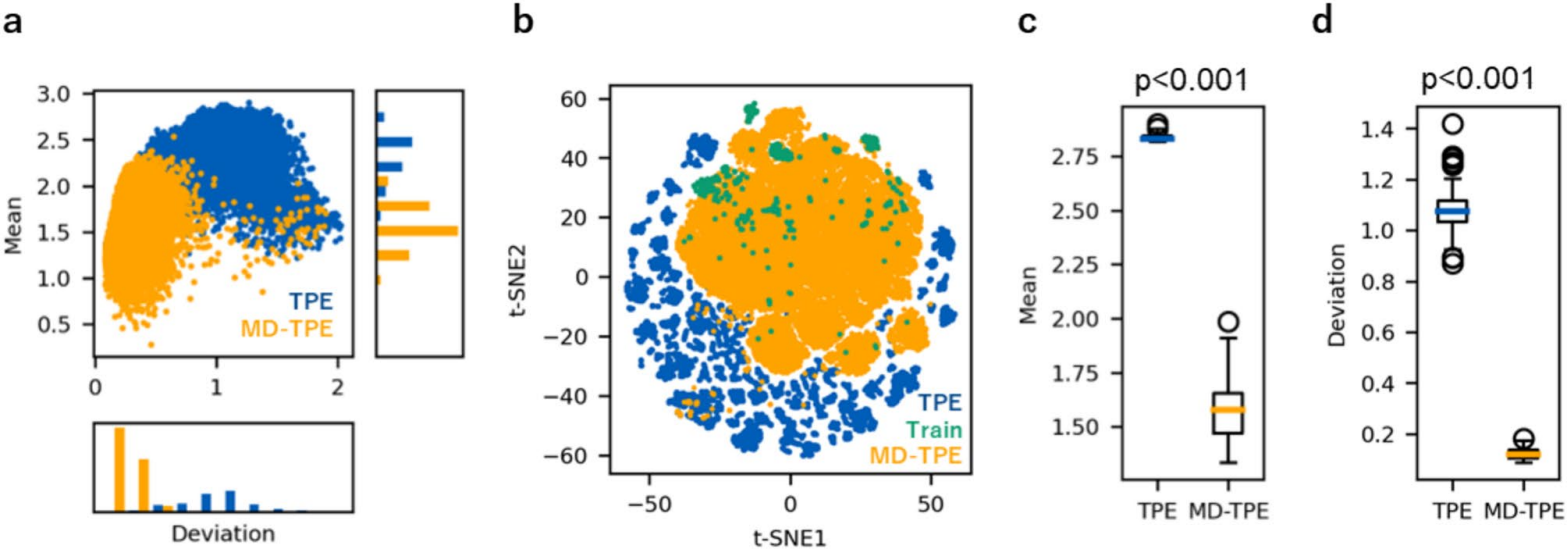
Results of the antibody affinity maturation task. (**a**) Relationship between the GP mean and GP deviation of the explored sequences from TPE and MD-TPE. (**b**) t-SNE visualization of the sequences using TAPE embedding. Blue, green, and orange dots represent sequences explored with TPE, the training set, and sequences explored with MD-TPE, respectively. (**c**) Distribution of the GP mean for the top 48 sequences. Mann-Whitney U test showed significant difference in median values between TPE and MD-TPE groups (p < 0.001). (**d**) Distribution of the GP deviation for the top 48 sequences. Mann-Whitney U test showed significant difference in median values between TPE and MD-TPE groups (p < 0.001).

We evaluated the performance of MD-TPE using the anti-MarvelD3 antibody data acquired in-house for anti-MarvelD3 antibody binding affinity measurements (see Binding affinity measurements of anti-MarvelD3 antibody in the Methods section). We examined whether MD-TPE could sample sequences with reliable predictive value. The antibody sequence of anti-MarvelD3 was optimized by MD-TPE to obtain high-binding-affinity sequences (see details in Antibody affinity maturation task in the Methods section). As shown in Fig. 3a, TPE sampled sequences have a high GP mean and deviation, whereas MD-TPE sampled sequences mainly with a lower deviation. Among the top 48 sequences based on the objective value, TPE exhibited a higher GP mean and deviation than MD-TPE (Figs. 3b and 3c, Supplementary Figure S4). In a low-dimensional space using t-SNE, sequences sampled by TPE were distant from the training data, whereas those sampled by MD-TPE were close to the training data (Fig. 3d). These results show that TPE explores unreliable regions with high deviation, while MD-TPE explores safe regions with low deviation. In summary, while TPE can explore sequences with a high GP mean, these sequences may lose functions such as expression and binding affinity because of their high deviation. In contrast, MD-TPE can explore sequences with a low deviation. The next section discusses the expression and binding affinity of the top 48 sequences from each objective function tested experimentally to evaluate the effect of safe exploration.

### Experimental evaluation of anti-MarvelD3 antibody using MD-TPE

**Fig. 4:**
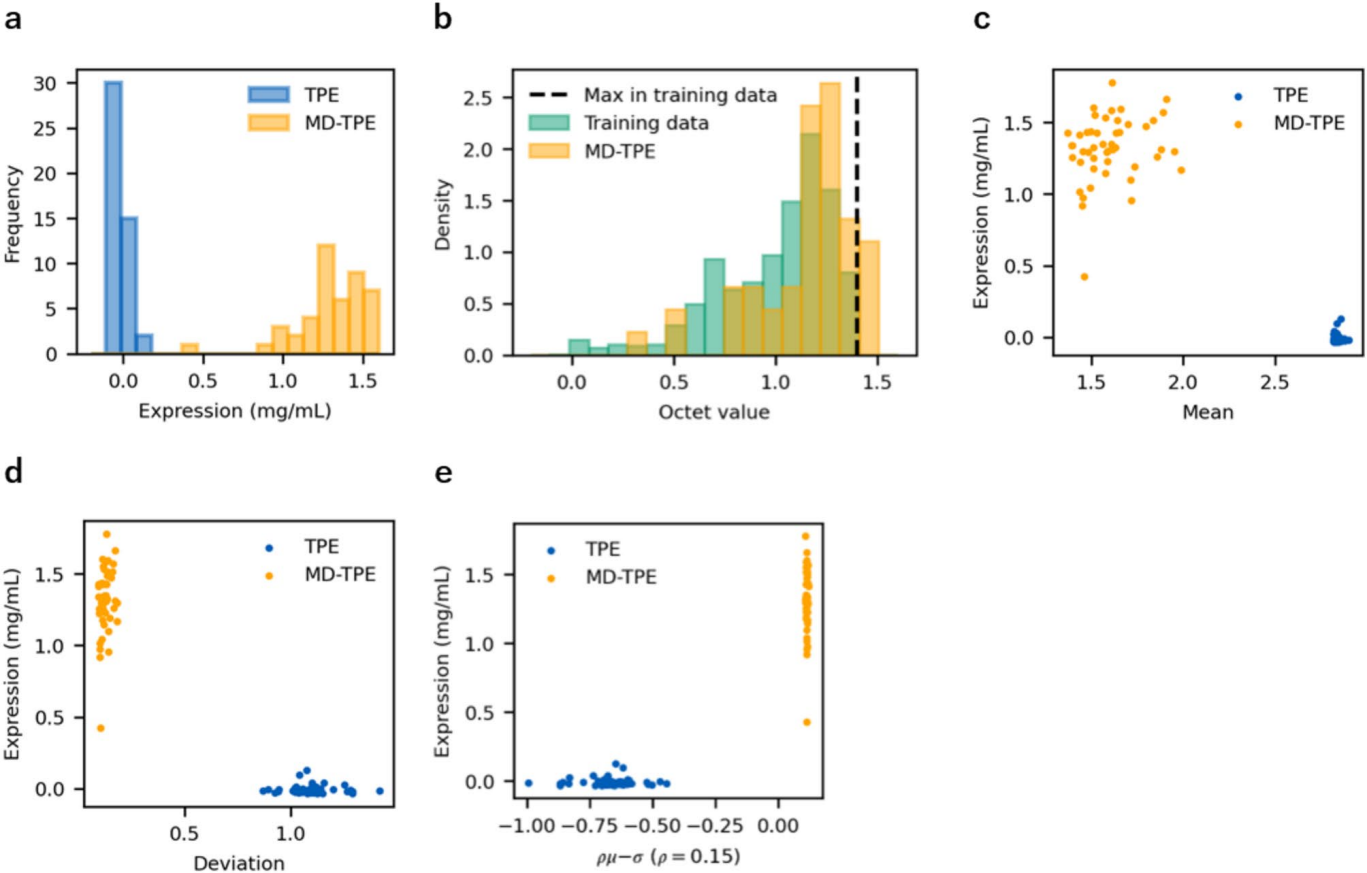
Results of antibody binding affinity and expression. (**a**)Distribution of expression of the top 48 sequences from TPE and MD-TPE. Mann-Whitney U test showed significant difference in median values between TPE and MD-TPE groups (p < 0.001). (**b**) Distribution of binding affinity of the training data and the top 48 sequences from MD-TPE. Mann-Whitney U test showed significant difference in median values between Training data and MD-TPE groups (p < 0.001). (**c**) Relationship between expression values and the GP mean. (**d**) Relationship between the expression values and GP deviation. (**e**) Relationship between the expression values and MD.

The top sequences predicted to have high binding-affinity by TPE and MD-TPE were experimentally verified for both binding affinity and expression. Figure 4a shows that almost all of the top 48 sequences sampled from MD-TPE were successfully expressed. In contrast, the top 48 sequences sampled from TPE had low expression levels (Fig. 4a). We could not measure the binding affinities of these sequences because of their low expression. Figure 4b shows that the sequences sampled from MD-TPE were evaluated by measuring the octet values for binding affinity (see Binding affinity measurements of the anti-MarvelD3 antibody in the Methods section). Among the 48 sequences, five showed octet values higher than the upper limit of the training set. Compared with the distribution of the training data, the distribution of sequences from MD-TPE tended to have higher octet values (Fig. 4b). Sequences with a high GP mean showed low expression, whereas those with a low predicted mean showed high expression (Fig. 4c). Moreover, sequences with high GP deviations showed low expression, whereas those with low GP deviations showed high expression (Fig. 4d). Sequences with high MD showed highly expression, whereas those with a low MD showed low expression (Fig. 4e).

### Advantage of safe exploration on antibody expression

In this section, we highlight the advantages of antibody expression using our proposed MD-TPE method. As shown in Fig. 4b, the antibodies from MD-TPE were highly expressed, whereas those from TPE were not. In contrast, antibodies predicted by TPE to have high binding affinities did not exhibit strong expression (Fig. 4c). This suggests that while the GP mean directly predicts binding affinity, it does not factor in the expression level. As shown in Fig. 4d, antibodies from TPE that had a high deviation were not expressed, and those from MD-TPE that had a low deviation were well expressed. This indicates that antibodies far from the training sequences were not expressed, whereas those near the training data were expressed well. This phenomenon is frequently observed in protein and antibody engineering. Given the limited space for antibody sequences that can be expressed, it is necessary to limit them to the vicinity of the training data. To achieve this, it is essential to incorporate GP deviation to efficiently identify functional antibodies with strong expression. By using MD as the objective function of TPE, the sampler can avoid sequences with large deviations. As shown in Fig. 4d, sequences with a high deviation exhibited low expression, whereas sequences with a low deviation exhibited high expression. These results suggest that samples with a high GP mean and deviation are unreliable. In addition, as shown in Fig. 4e, sequences with high MD exhibited high expression, whereas those with low MD exhibited low expression. This result indicates that MD indirectly factors expression levels, which is crucial in antibody engineering.

### Robustness analysis of protein embedding models

One factor that affects the performance of the proposed framework is which protein’s LLM-based embeddings are used as features. Therefore, we evaluated the performance using fivefold cross-validation on the GFP brightness task and anti-MarvelD3 antibody proxy model creation data for features from several embedding models besides TAPE. In the GFP brightness task, ProtT5 showed low accuracy, while TAPE performed the best. However, no substantial differences were observed between TAPE, ESM-1, and ESM-2 (Supplementary Table 1). For the anti-MarvelD3 antibody task, although ESM-1 showed the best performance, the performance variations among all models were minimal, with all metrics falling within similar ranges (Supplementary Table 2).

### Related work

Several related studies have explored methods for limiting the search space to the training data. In the framework of offline MBO, conservative objective models (COMs) have been proposed^13^ wherein a regularization term was introduced in the objective to prevent distributional shifts. They aimed to regularize the model using adversarial examples by penalizing out-of-distribution data. In the framework of adaptive sampling, conditioning by adaptive sampling (CbAS) has been proposed^12^ where an importance weight between the proposed distribution and the training data was introduced in the Monte Carlo approximation for the objective for obtaining samples close to the training data by weighting their importance.

While our motivation is to demonstrate the feasibility of sequence design through wet-lab experiments on antibodies by incorporating Gaussian process predictive deviation into the TPE-based MBO framework, comparing our approach with these methods remains an important future task. As a preliminary experiment, we conducted a simple comparison between our proposed method and CbAS to validate our approach (Supplementary Figure S5). However, a more comprehensive comparison with other methods requires further investigation to understand how differences between methods—such as mutation-based versus de novo design approaches, and the use of one-hot encoding versus PLM embedding—affect performance.

Furthermore, previous studies have used mean variance and conditional value at risk (CVaR) for the objective function in online Bayesian optimization and bandit problem^32,33^. These studies leveraged the uncertainty from a learned model as the variance. The motivation for these studies was different from that of our study.

## Conclusion

We proposed a safe Model-Based Optimization (MBO) for protein sequence design by introducing uncertainty into the objective function. In the two tasks we examined (one synthetic task using public GFP data and one real antibody design application), our method effectively identified solutions in the vicinity of the training data, which were in a reliable region. Experimental verification of the anti-MarvelD3 antibody showed that MD-TPE ensured safe exploration and prevented the selection of samples far from the training data. In contrast, TPE selected samples far from the training data and clones were not expressed. Our approach was able to generate sequences with higher affinities than the training data in wet lab experiments, although the sequences we could experimentally verify represent only a very small subset of the vast antibody design space.

Despite these promising results, our approach has several limitations. We have only validated our approach on two specific tasks, and its generalizability to other protein engineering problems requires further investigation. Additionally, we lack direct comparisons with related methods, which makes it difficult to quantify performance advantages over existing approaches. These initial results nevertheless provide insights that our method can accelerate the identification of desirable sequences in protein or antibody engineering, and addressing these limitations presents important directions for future work.

## Methods

### Tree-structured Parzen estimator (TPE)

The tree-structured Parzen estimator, proposed by Bergstra et al.^21^, is an advanced method for black box optimization that combines Bayesian optimization with Parzen window density estimation. The key advantage of TPE is its ability to handle categorical variables naturally, which enables its application in biological sequence design tasks.

The TPE algorithm seeks to maximize the expected improvement (EI) in the objective function. Let X represent the search space and Y represent the corresponding performance scores and x represent sequence. In our offline molecular optimization case, X and Y are combinations of discrete variables and the output of the proxy function, respectively. Given a threshold γ, the algorithm divides the observed input $x\in X$ into two sets:

$${\text{Set for }"\text{good}"\text{ sequences}:X}_{good}=\left\{x\in X\right|y\left(x\right) \le\upgamma \}$$

$${\text{Set for }"\text{bad}"\text{ sequences}:X}_{bad}=\left\{x\in X\right|y\left(x\right) >\upgamma \}$$

The TPE sampler attempts to sample $x$ that maximizes the EI, which is defined as the expectation of improvement in the objective function value at a given candidate configuration x, considering the previously observed configurations:

$$EI\left(x\right)= E\left[max\left(0, f\left({x}_{best}\right)- f\left(x\right)\right)\right],$$

Bergstra et al.^21^ demonstrated that the EI is proportional to the likelihood ratio of $p\left(x|{X}_{good}\right)/p(x|{X}_{bad})$.

$$EI\left(x\right) \propto p\left(x|{X}_{good}\right)/p(x|{X}_{bad})$$

Therefore, to maximize EI(x), the TPE sampler selects the next candidate configuration ${x}^{*}$ that maximizes the ratio $p\left(x|{X}_{good}\right)/p(x|{X}_{bad})$:

$${x}^{*}=argma{x}_{\left\{x\in X\right\}}(p\left(x|{X}_{good}\right)/p(x|{X}_{bad}))$$

In this way, TPE can be used to select the appropriate amino acids to be placed at each mutation position by dividing observations into two groups—“good” and “bad”—and estimating their density ratio.

The output of the proxy function was set as the objective score for the TPE. For the TPE sampler search space, sets of candidate amino acids for each mutated position were provided. The Optuna^34^ implementation was used to execute the TPE sampler. Detailed parameter settings are described in each section of the task.

### GFP brightness task

Green fluorescent protein (GFP) is widely used in medical and biological research, and the creation of bright GFPs is a goal in the field of bioengineering. Sarkisyan et al.,^25^ provided an original dataset comprising brightness measurements from modified GFP. The dataset contains information on 56,086 GFP sequences and their brightness and has been used to benchmark the performance of model-based optimization in several research^11,12,35^. We used the dataset provided by Trabacco et al.^35^. The distributed representation, with size ${x}_{embed}\in {\mathbb{R}}^{756},$ for each GFP sequence was obtained with a pretrained protein language model (PLM) called Tasks Assessing Protein Embeddings (TAPE) method^22^. As a proxy model, we trained the GP model as follows: GFP sequences with two or fewer modifications from the parent sequence avGFP were used as training data (Supplementary Figure S6), and the number of training data points was 1128. This procedure intended to mimic the practical molecular optimization process, because we usually begin with sequences with a few residue substitutions from the parent sequence. As a pseudo-oracle, LightGBM^36^ was trained with all the data. We used this pseudo-oracle model as a surrogate for true GFP brightness according to a previous study^35^. We used the trained GP model as the proxy function. To construct this GP model, we used a combination of Matérn and linear kernels.

The positions and residues corresponding to the top 30 brightness values were selected as the mutated positions and residues to define the search protein sequence space. This sequence space consisted of 25 mutated positions, and all possible combinations of mutations reached more than 188 million. All mutated positions and residues are summarized in Supplementary Table 3. The top 10 sequences in the training dataset were used for warm start initialization. We set the trial number 3000 and used the default parameters set in the Optuna framework. We set the risk tolerance parameter ρ to 0.15 based on empirical evaluation. To determine this value, we conducted a grid search over several candidate values (ρ ∈ {0.05, 0.10, 0.15, 0.20, 0.30, 0.40}) and evaluated each by running brief TPE optimizations. For each candidate value, we analyzed whether the resulting optimized sequences maintained GP deviation levels comparable to those observed in our training data of the proxy model. This approach was designed to mitigate out-of-distribution issues by ensuring that the optimization process would not favor sequences with uncertainty levels significantly higher than those in our training distribution.

### Antibody affinity maturation task

In this task, we aimed to identify an antibody that has a higher affinity for the antigen MarvelD3, which is a tight junction protein with a four-span transmembrane. We applied MD-TPE to obtain anti-MarvelD3 antibodies with higher affinities. We employed GP as a proxy model to estimate the uncertainty and embedded antibody sequences using TAPE, similar to the GFP brightness task. The positions and residues corresponding to the top 100 sequences with high affinities were selected as mutated positions and residues to define the search protein sequence space. This sequence space consisted of 42 mutated positions, and all possible combinations of mutations reached more than $4.47\times {10}^{18}$. All the mutated positions and residues are summarized in Supplementary Table 4. The top 10 sequences in the training dataset were used for warm start initialization. We set the trial number to 5000 and used the default parameters set in the Optuna framework. We set the risk tolerance parameter ρ to 0.15, as in the GFP brightness task.

### Scalability of gaussian process regression for high-dimensional protein embeddings

While Gaussian process regression can become computationally expensive in high-dimensional spaces, we found that our implementation scaled to the high-dimensional embeddings used in this study. Without using approximation methods such as sparse GPs or random feature expansions, we applied the standard GP regression model directly. For the GFP brightness task, we created a proxy model using 758-dimensional features obtained by embedding with TAPE. The computational performance was reasonable, with an execution time of approximately 67 s, average memory usage of about 711 MB, and memory fluctuation range of 96 MB. For the anti-MarvelD3 antibody task, we created a proxy model using 1516-dimensional features obtained by embedding heavy chain and light chain separately with TAPE. The execution time was approximately 384 s, with an average memory usage of about 810 MB, and memory fluctuation range of 127 MB. These resource requirements are Intel(R) Xeon(R) Gold 6230R CPU @ 2.10 GHz (52 cores, 4.0 GHz max).

### Binding affinity measurements of anti-MarvelD3 antibody

Antibodies were transiently expressed as previously reported^37^. Briefly, plasmids encoding the designed heavy or light chains were prepared, and the recombinant antibodies were expressed transiently using a 1 mL culture of Expi293F TM cells (Thermo Fisher, Cat. No. A14527) according to the manufacturer’s instructions. The antibodies were captured using protein A (GL Science, Cat No.7510-H0165) from the supernatants and eluted into 0.2 mL of buffer. The concentration of the antibody in the buffer was determined by measuring absorbance at 280 nm. To obtain the MarvelD3/CD3 bispecific antibody, anti-MarvelD3 and anti-CD3 antibodies were mixed and gently reduced in a buffer. Selective HC heterodimerization was achieved by inducing charge repulsion between the HC. Ion-exchange chromatography confirmed that the resulting antibody was eluted between the two parental antibodies, indicating that the antibody was prepared as intended.

The binding ability of each bispecific antibody was measured using the Octet HTX system (Sartorius). Extracellular vesicles displaying CD81 (BioLegend, 349,514) and human MarvelD3 antigens were captured on the SAX sensor chip using a biotinylated anti-CD81 antibody. Subsequently, a 600 s baseline step in PBS with 0.1% BSA was performed, followed by the measurement of the association and dissociation responses of 20 nM antibodies in the same buffer for 900 s and 1500 s, respectively. The affinity of each antibody was represented as a wavelength shift (nm) between the baseline and end of the association phase. The baseline, association, and dissociation phases of the measurement were performed at 30 °C and shaking at 1,000 rpm. A representative antibody was included as a positive control in each measurement batch. Although individual samples were measured only once, this control antibody was repeatedly measured across all sessions, yielding a coefficient of variation (CV) of 15.9%.

## Supplementary Information

Supplementary Information.

## Data Availability

The public GFP dataset is available from the design-bench repository (https://github.com/brandontrabucco/design-bench).
