# Supplementary material for: Safe model based optimization balancing exploration and reliability for protein sequence design
Source: Sci Rep. 2025 Jul 29;15:27568. doi: 10.1038/s41598-025-12568-5 (PMC12307608; doi:10.1038/s41598-025-12568-5)
Supplement: Supplementary file 1 — Supplementary Information. [file 41598_2025_12568_MOESM1_ESM.docx]

**Supplementary figure S1**

**
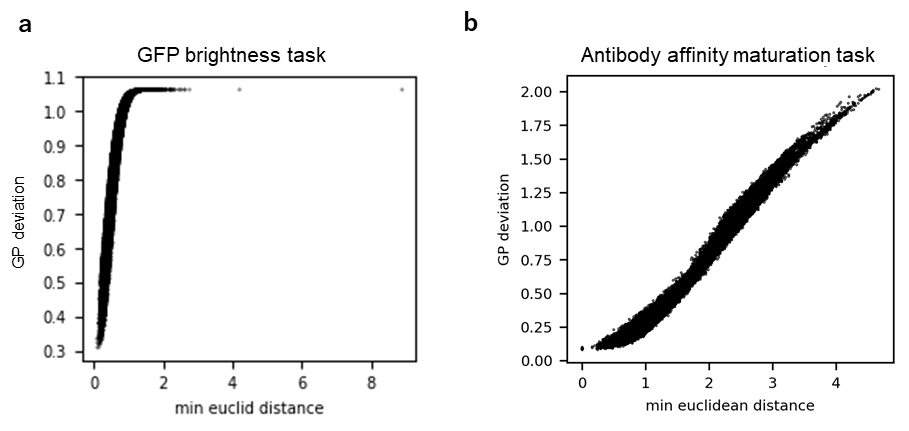
**

Supplementary figure S1. correlation between GP variance and the embedding-space distance from known functional sequences.

a) Scatter plot showing correlation between the Euclidean distance from the nearest sequence in the Proxy model training data and the GP deviation for data explored by TPE and MD-TPE in the GFP brightness task. b) Scatter plot showing correlation between the Euclidean distance from the nearest sequence in the Proxy model training data and the GP deviation for data explored by TPE and MD-TPE in the Anti-MarvelD3 antibody affinity maturation task.

**Supplementary figure S2**

**
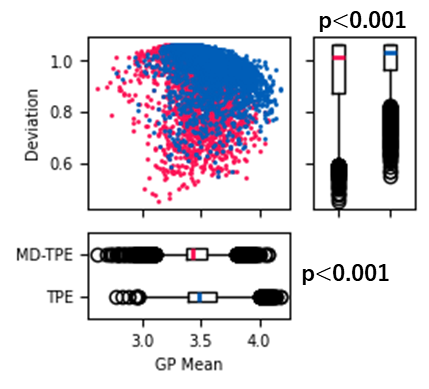
**

Supplementary Figure S2. The relationship between the GP mean and deviation of the sampled value in the GFP brightness task.

For the GP mean on the vertical axis, Mann-Whitney U test showed significant difference in median values between TPE and MD-TPE groups (p < 0.001). For the GP deviation on the horizontal axis, Mann-Whitney U test showed significant difference in median values between TPE and MD-TPE groups (p < 0.001).

**Supplementary figure S3**

**
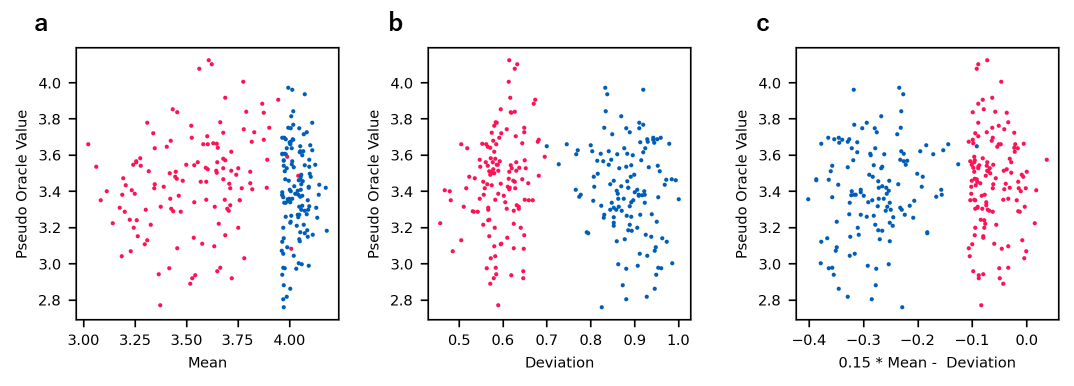
**

Supplementary Figure S3. Pseudo-oracle values in the GFP brightness task.

1. Relationship between the pseudo-oracle values and GP mean. b) Relationship between the pseudo-oracle values and GP mean. c) Relationship between the pseudo-oracle values and MD.**Supplementary figure S4**

**
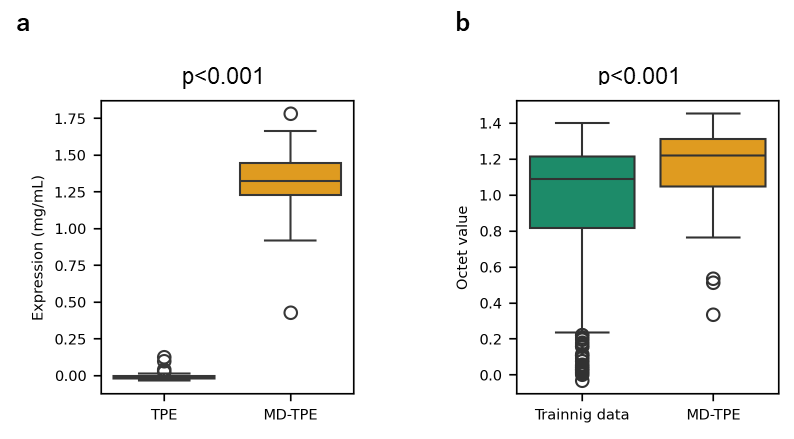
**

Supplementary figure S4. Results of antibody binding affinity and expression.

a) Distribution of expression of the top 48 sequences from TPE and MD-TPE. Mann-Whitney U test showed significant difference in median values between TPE and MD-TPE groups (p < 0.001). b) Distribution of binding affinity of the training data and the top 48 sequences from MD-TPE. Mann-Whitney U test showed significant difference in median values between Training data and MD-TPE groups (p < 0.001).

**Supplementary figure S5**


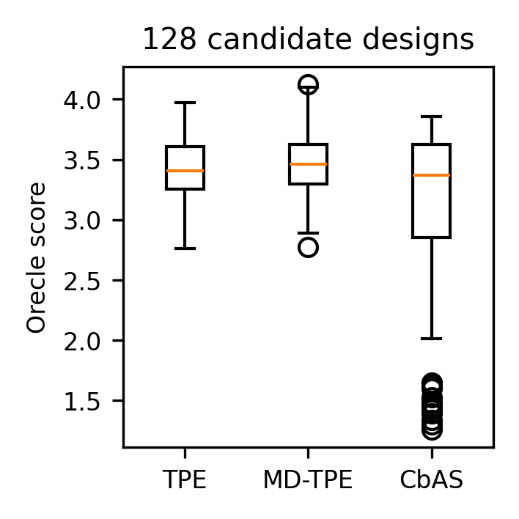


Supplementary figure S5. Comparison of the 100th percentile score of 128 candidate designs between CbAS, MD-TPE, and TPE in the GFP task".

In the GFP brightness task, when comparing Oracle scores for 128 candidate designs, there was no substantial difference in median scores across methods. However, designs generated by CbAS exhibited a tendency toward greater performance variability compared to other methods. The parameter settings for MD-TPE and TPE are described in detail in the Methods section under GFP brightness task. For CbAS, we used the default parameter values.

**Supplementary figure S6**

**
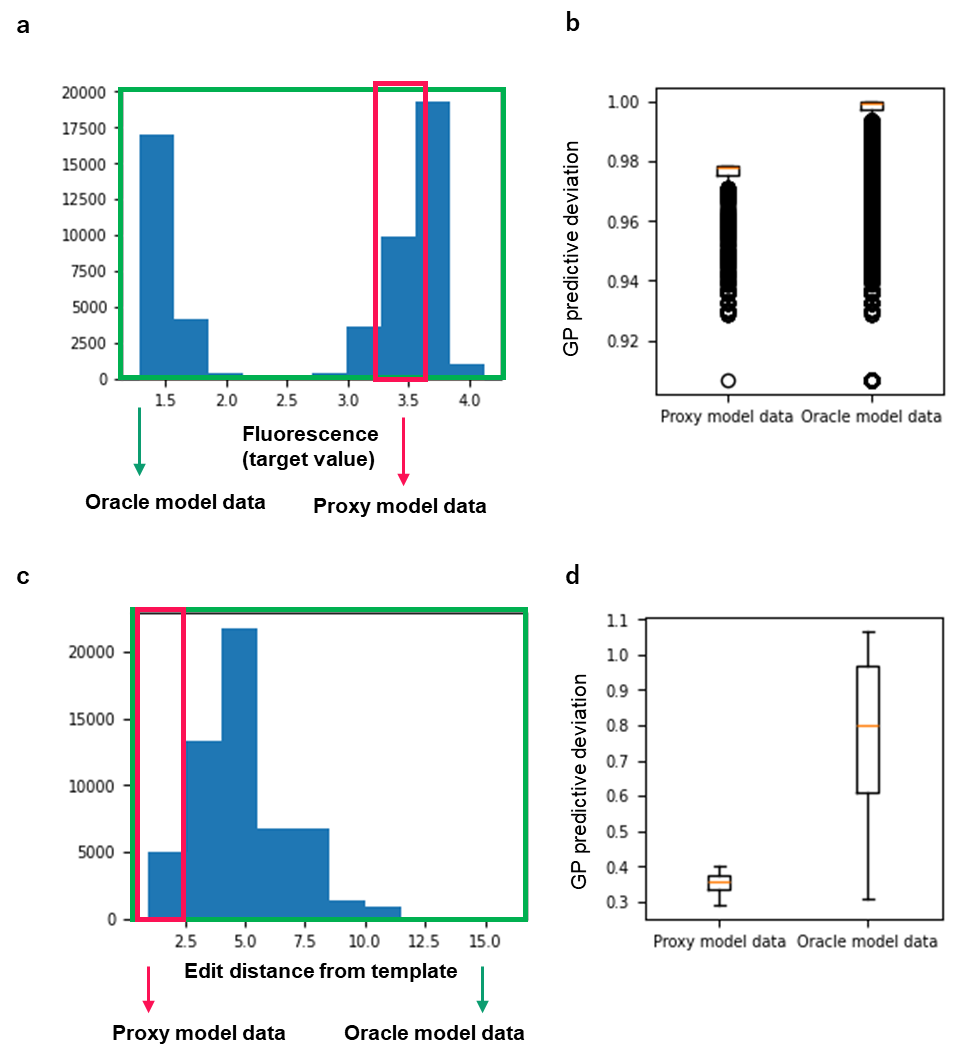
**

Supplementary Figure S5. Changes in GP predictive deviation due to different data splits in the GFP brightness task

a) Data used for constructing proxy model and oracle model in previous studies. Data between the 50-60th percentile of Target values in the GFP task was used for training the proxy model, as indicated by the red frame. The green frame shows all data, which represents data for training the oracle model that indicates the ground truth of the GFP offline MBO task. b) GP predictive deviation of the data used for the proxy model and the oracle model. c) Data used for constructing proxy model and oracle model in this study. Data with Edit distance < 2 from the template sequence in the GFP task was used for training the proxy model, as indicated by the red frame. The green frame shows all data, which represents data for training the oracle model that indicates the ground truth of the GFP offline MBO task. d) GP predictive deviation of the data used for the proxy model and the oracle model.

The evaluation method using GFP in existing research involved data where the proxy model's training data and search space (oracle model’s training data) were close, making it difficult for out-of-distribution problems to occur. Therefore, we modified the task. In the evaluation method of existing research, there was little difference in predictive deviation between the oracle model data (ground truth) and the proxy model's training data, indicating they were not out-of-distribution (Supplemental Figure S5 a-b). Therefore, by limiting the selection method for proxy model training data to those with small Edit distances from the template, we created a difference in predictive deviation between the proxy model data and oracle model data (Supplemental Figure S5 c-d), causing out-of-distribution problems when executing offline MBO.

**Supplementary Table 1**

| Embedding | MSE | R² |
| --- | --- | --- |
| TAPE | 0.2745 ± 0.0345 | 0.4528 ± 0.0387 |
| ESM-1 | 0.2837 ± 0.0278 | 0.4327 ± 0.03980 |
| ESM-2 | 0.2904 ± 0.0387 | 0.4186 ± 0.0713 |
| ProtT5 | 0.5008 ± 0.0322 | -0.00155 ± 0.0017 |

**Supplementary Table 2**

| Embedding | MSE | R² |
| --- | --- | --- |
| TAPE | 0.0291 ± 0.0028 | 0.653 ± 0.030 |
| ESM-1 | 0.0273 ± 0.0022 | 0.675 ± 0.027 |
| ESM-2 | 0.0284 ± 0.0029 | 0.662 ± 0.027 |
| ProtT5 | 0.0275 ± 0.0029 | 0.671 ± 0.036 |

**Supplementary Table 3**

| mutated position | included residues |
| --- | --- |
| 2 | A, G |
| 28 | C, S |
| 37 | N, S, Y |
| 40 | L, M |
| 42 | L, M |
| 60 | P, T |
| 71 | R, L |
| 73 | H, P |
| 103 | N, C, H, I, Y |
| 108 | A, S |
| 109 | Q, E |
| 126 | I, L |
| 135 | L, M |
| 142 | N, G |
| 156 | G, K, V |
| 161 | A, V |
| 162 | N, T |
| 173 | S, T |
| 176 | L, V |
| 187 | C, G |
| 189 | G, V |
| 201 | I, T |
| 204 | A, V |
| 229 | H, Y |
| 232 | D, Y |

**Supplementary Table 4**

| mutated position | included residues |
| --- | --- |
| VH27 | F, Y |
| VH28 | R, T |
| VH29 | F, Y |
| VH31 | G, T |
| VH32 | A, D, G, M, Q, R, S, T, W |
| VH34 | I, M |
| VH50 | I, T |
| VH51 | I, K, M, Q |
| VH54 | A, G, W |
| VH58 | F, Y |
| VH59 | F, Y |
| VH60 | A, S |
| VH61 | A, E, S |
| VH62 | T, W |
| VH63 | A,D |
| VH64 | A, F, I, K, P, Q |
| VH65 | E, G, S |
| VH84 | A,D |
| VH96 | A, L |
| VH101 | A, T |
| VH102 | E, I, Q, T |
| VH105 | Q, R |
| VH108 | L, M, T |
| VL24 | D, E, Q |
| VL25 | A, L |
| VL26 | D, E,P, S |
| VL27 | E, K, Q |
| VL28 | R, S |
| VH30 | R, S, W |
| VH31 | G, K, R, S |
| VL53 | E, K, L, R, T |
| VL54 | E, L |
| VL55 | A, I |
| VL56 | D, E, P, S |
| VL89 | M, Q, S, T, V, W, Y |
| VL92 | K, Q, Y |
| VL93 | E, F |
| VL95 | A, S |
| VL95a | H, S, W, Y |
| VL95d | D, E, H, I, R, S, V |
| VL100 | G, P, Q |
| VL105 | D, E |
